# A proof-of-concept study for patient use of open notes with large language models

**DOI:** 10.1093/jamiaopen/ooaf021

**Published:** 2025-04-09

**Authors:** Liz Salmi, Dana M Lewis, Jennifer L Clarke, Zhiyong Dong, Rudy Fischmann, Emily I McIntosh, Chethan R Sarabu, Catherine M DesRoches

**Affiliations:** Department of Women’s and Children’s Health, Uppsala University, 752 37 Uppsala, Sweden; OpenNotes, Beth Israel Deaconess Medical Center, Boston, MA 02215, United States; #OpenAPS, Seattle, WA 98101, United States; Department of Neurological Surgery, University of California, San Francisco, CA 94117, United States; OpenNotes, Beth Israel Deaconess Medical Center, Boston, MA 02215, United States; Richmond, VA 23120, United States; Guelph, ON N1E 5J5, Canada; OpenNotes, Beth Israel Deaconess Medical Center, Boston, MA 02215, United States; Jacobs Technion-Cornell Institute, Cornell Tech, New York, NY 10044, United States; OpenNotes, Beth Israel Deaconess Medical Center, Boston, MA 02215, United States; Harvard Medical School, Boston, MA 02115, United States

**Keywords:** generative AI, open notes, large language models, patient portals

## Abstract

**Objectives:**

The use of large language models (LLMs) is growing for both clinicians and patients. While researchers and clinicians have explored LLMs to manage patient portal messages and reduce burnout, there is less documentation about how patients use these tools to understand clinical notes and inform decision-making. This proof-of-concept study examined the reliability and accuracy of LLMs in responding to patient queries based on an open visit note.

**Materials and Methods:**

In a cross-sectional proof-of-concept study, 3 commercially available LLMs (ChatGPT 4o, Claude 3 Opus, Gemini 1.5) were evaluated using 4 distinct prompt series—*Standard*, *Randomized*, *Persona*, and *Randomized Persona*—with multiple questions, designed by patients, in response to a single neuro-oncology progress note. LLM responses were scored by the note author (neuro-oncologist) and a patient who receives care from the note author, using an 8-criterion rubric that assessed *Accuracy*, *Relevance*, *Clarity*, *Actionability*, *Empathy/Tone*, *Completeness*, *Evidence*, and *Consistency*. Descriptive statistics were used to summarize the performance of each LLM across all prompts.

**Results:**

Overall, the Standard and Persona-based prompt series yielded the best results across all criterion regardless of LLM. Chat-GPT 4o using Persona-based prompts scored highest in all categories. All LLMs scored low in the use of *Evidence*.

**Discussion:**

This proof-of-concept study highlighted the potential for LLMs to assist patients in interpreting open notes. The most effective LLM responses were achieved by applying *Persona*-style prompts to a patient’s question.

**Conclusion:**

Optimizing LLMs for patient-driven queries, and patient education and counseling around the use of LLMs, have potential to enhance patient use and understanding of their health information.

## Introduction

Artificial intelligence (AI) tools, including large language models (LLMs), have garnered interest as a means to advance clinical research and reduce clinician workload in healthcare settings.^1–5^ Following the COVID-19 pandemic, and the enforcement of the 21st Century Cures Act Information Blocking rule, health systems with patient portals experienced a surge in patient-initiated messages and increasing clinician workload.^6–10^ Clinicians raised concerns about the effect increased messaging had on physician burnout.^11^ In response, some health systems began charging patients for advice provided through the portal,^12^ while others worried about potential ethical ramifications of charging fees for message exchange.^13^ Researchers have begun deploying and testing AI tools to alleviate the burden of messaging and explore potential benefits of AI for clinicians.^14^ One study found that patients perceived responses from an AI chatbot as more empathetic and comprehensive than those from their clinicians.^14^ A separate study found chatbot responses to diverse medical queries were largely accurate, with improvements over time.^15^

Although patient uses of health AI have not been as extensively covered in academic journals as clinician use cases, patients are also adopting AI tools to understand their health.^16^ A recent KFF poll found that 17% of adults in the United States said they used a chatbot at least once a month to find information about their health,^16^ and individuals with chronic conditions report using commercial LLMs to augment their “thinking and decision-making” when facing medical challenges.^17^

Patient use of their health information to gain understanding and make decisions about their care, and the care of loved ones, is not a new idea. Over the prior decade, research in the domain of “open notes” demonstrated that when patients have access to their progress notes, they felt more engaged in care,^18^ were more likely to take medications as prescribed,^19^ and shared notes with their care partners.^20^ Patients who read their notes tend to ask more informed questions, however, notes can be confusing for some patients.^21^ LLMs have the potential to help with this confusion.

While there is an increasing evidence base being developed around the ethical, legal, regulatory, and technical deployments of generative AI at the health systems level,^22–28^ the effectiveness and reliability of LLMs for responding to queries based on open notes remain largely unexplored from the patient point of view. Much of the research surrounding generative AI in healthcare has focused on clinician-centric applications.^3^ There are few examples of AI being evaluated specifically to assist patients in managing the cognitive burdens of understanding complex medical information or facilitating their care,^29^ including care outside of hospital or clinic walls.^30^

Rather than relying solely on clinician-generated queries,^15^ in this proof-of-concept study we attempted to address this gap by evaluating the reliability and accuracy of 3 commercially available chat-based LLMs by designing prompts that reflect on the lived experiences of patients with brain tumors in answering a series of patient-generated questions about a real neuro-oncology progress note (open note). The goal of this work was to assess the effectiveness and versatility of LLMs in responding to patient queries based on clinical notes, considering various prompt styles, as well as performance across models, from the perspective of both a patient and a neuro-oncology clinician. By leveraging a structured rubric to assess responses across different models and prompts, this study highlights the resulting differences in LLMs, and the role prompts may play for patients, when used in conjunction with open notes.

## Methods

### Study design

This cross-sectional proof-of-concept study employed a multi-model, multi-scenario approach to assess the performance of 3 LLMs in responding to patient queries about a single clinical note written by a neuro-oncologist at an academic medical center. The note used for this analysis was accessed by a patient through their MyChart-based patient portal at the University of California, San Francisco (Epic Systems Corporation) (Appendix S1). The Beth Israel Deaconess Medical Center Community on Clinical Investigations determined this study is not human subjects research, and a signed identifiable patient statement was submitted to this journal.

The proof-of-concept study involved 4 series of questions designed to explore LLM adaptability to different questioning strategies and instructional framings (Table 1). Three commercially available LLMs were each tested to compare their effectiveness, receiving identical series of prompts, to ensure comparability: ChatGPT (Open AI, GPT4o accessed via API on May 22, 2024), Claude (AnthropicAI, Claude-3-Opus-20240229 accessed via API on June 4, 2024), and Gemini (Alphabet/Google, Gemini-1.5-pro accessed via API on June 4, 2024). The prompting approach included specific personas as well as variations on the order in which questions were asked.

**Table 1.** Large language model (LLM) prompt series and descriptions.

Prompt series	Description
Standard order	Patient questions were presented sequentially based on the order in which the information appeared in the clinical note (Appendix S1).
Randomized order	The same patient questions were presented in a randomized order.
Persona	Patient questions were presented sequentially based on the order in which the information appeared in the clinical note (matching the Standard Order series), but were prefixed with the Persona instruction, “*You are an expert oncologist who specializes in brain cancer*”
Randomized persona	The randomized order series, with the addition of the identical Persona instruction.

### Evaluation criteria and process

**Figure 1.**
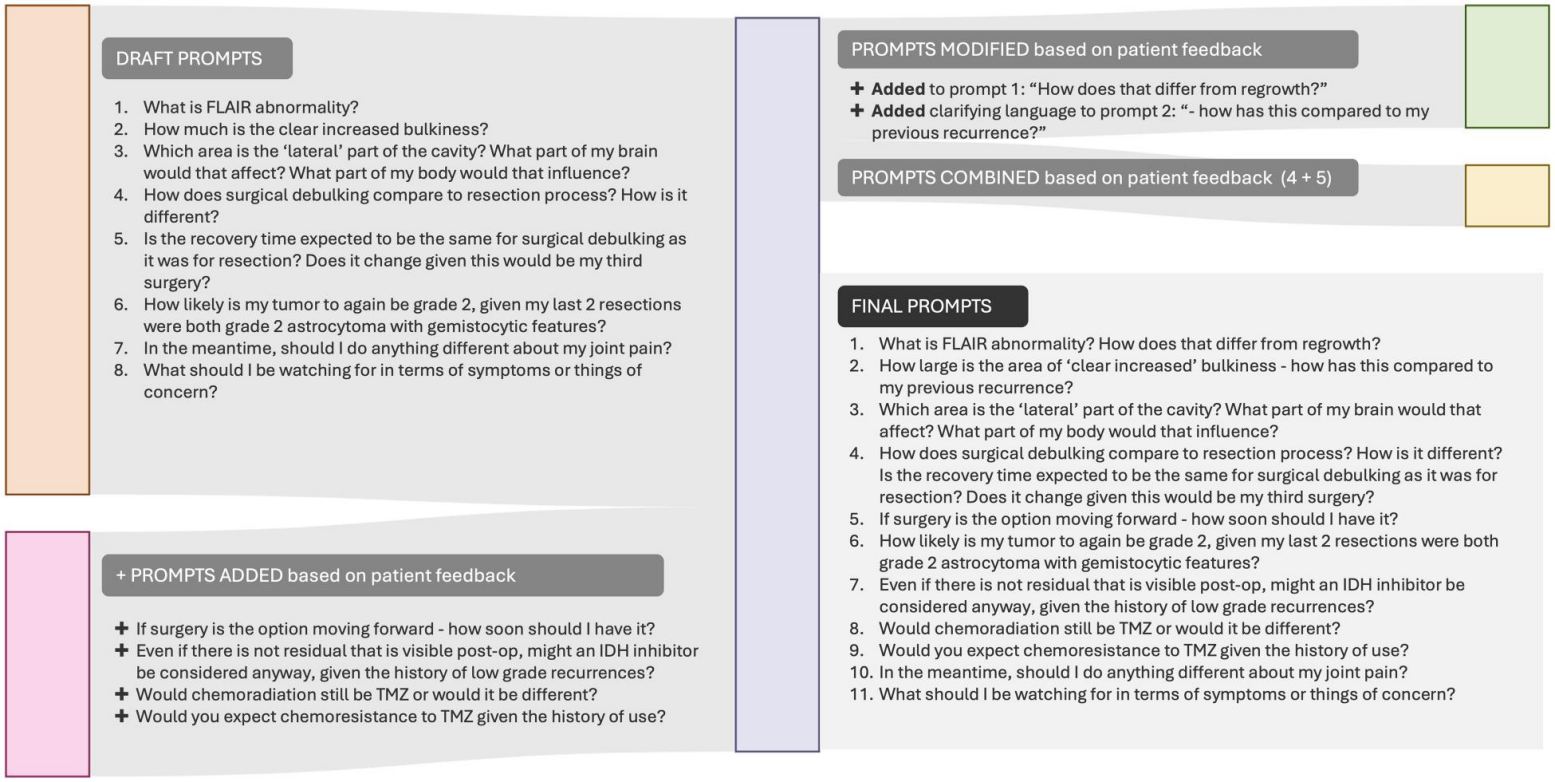
The prompt refinement process.

Each prompt series was evaluated as an independent test with each model, conducted in a simulated new chat (via the API, with system messaging instructions) with the LLM, ensuring no carryover of context or knowledge from previous interactions. No other custom instructions nor settings, such as memory features, were used. The prompts were first developed as a list of topics (D.L.), evaluated by a person with lived expertise of a brain tumor (L.S.), slightly revised (D.L.), and reviewed again by 2 additional individuals with brain tumors (R.F., E.M.), then finalized (D.L.) into each series (Appendix Table S2). (Figure 1 of the Prompt Refinement Process.)

A detailed rubric was developed to score LLM responses, which included: Accuracy of Medical Information (*Accuracy*), Relevance to the Patient Query (*Relevance*), Clarity of Communication (*Clarity*), *Actionability*, Empathy, and Tone (*Empathy/Tone*), *Completeness*, Reference to Evidence or Clinical Guidelines (*Evidence*), and Internal Consistency (*Consistency*) (ie, staying on topic and not losing detail). Each criterion was rated on a 1-5 scale, with detailed descriptors for each score provided to the patient and clinician evaluator. The rubric was first developed independently, then compared with the PDQI-9 (a rubric for assessing the quality of clinical notes, see Appendix Table S3).^31^

### Data collection

LLM responses were evaluated by a neuro-oncologist (J.C.) as well as a patient (L.S.) who receives care from the neuro-oncologist. The doctor/patient raters were blinded to which prompt series the response resulted from, as well as the LLM identity, in order to minimize bias. To further limit bias, a random order was assigned to the models and series of responses, to which the raters were blinded. The raters were encouraged to only review 3 series at a time to limit fatigue. A training session was conducted (by D.L.) to familiarize raters (J.C., L.S.) with the rubric and scoring process, which used Google Forms (Alphabet) for the blinded scoring process. Data was exported as CSV and analyzed using Python in a Jupyter Notebook.

### Statistical analysis

Descriptive statistics were employed to summarize the performance of each language model across all prompts. Normality was assessed using the Shapiro-Wilk test. We used ANOVA or the Kruskal-Wallis (for non-normal data) test to identify performance differences between models and prompt series. Post-hoc analyses using Dunn’s tests with Bonferroni correction for multiple comparisons were applied where applicable. Inter-rater reliability between the 2 raters (J.C., L.S.) was evaluated using Gwet’s AC1 and Krippendorff’s Alpha.^32^ Interaction effects between model type and prompt series were analyzed using 2-way ANOVA, and interaction plots were created to visualize these effects. Finally, a heatmap was generated to visualize the average scores for each LLM response across all performance metrics.

## Results

**Figure 2.**
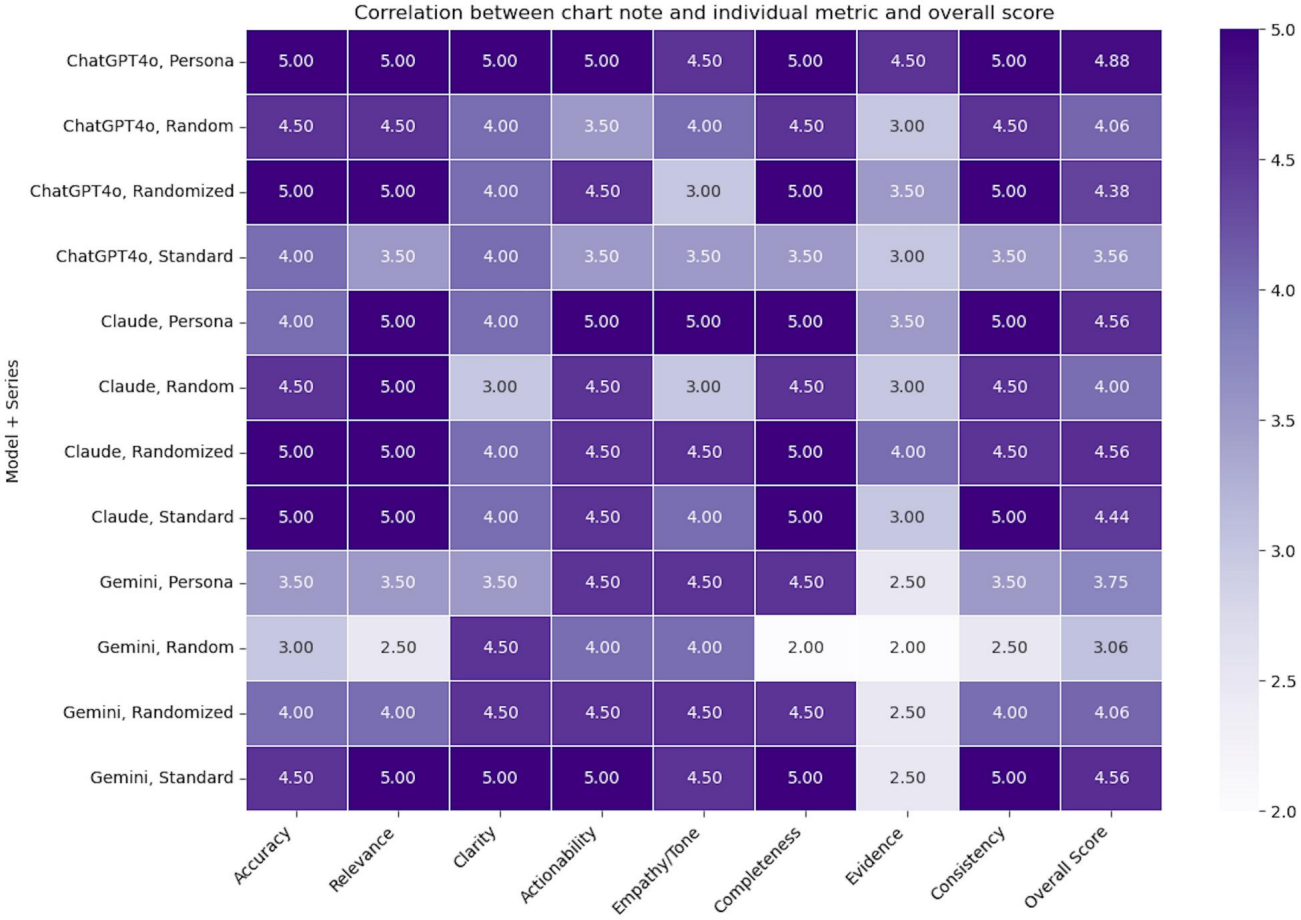
Correlation between LLM model type and prompt series, the average score for individual metrics and overall score.

In general, the Standard Order and Persona series performed well (Figure 2 and Appendix Figure S7 and Tables S4-1 and S4-2 for score data) in this proof-of-concept study. The mean score for LLM outputs for Claude-Standard, Claude-Persona, Gemini-Standard, and ChatGPT4o-Persona was between 4.00 and 5.00 across 8 of 9 metrics—indicating a consistent performance by delivering accurate, relevant, and clear responses. Mean scores were lowest for these prompt/model combinations for the use of *Evidence*. There was a variation in *Empathy/Tone* scores across LLM outputs, with some, like Claude-Persona and ChatGPT4o-Persona, scoring higher. LLM outputs for Gemini-Standard and ChatGPT4o-Persona excelled in *Actionability* and *Completeness* (Appendices S4-1 and S4-2).

The post-hoc Dunn’s test revealed significant differences in the *Accuracy* scores between Gemini and Claude (*P* = .028) and Gemini and ChatGPT4o (*P* = .022), as well as significant differences in *Relevance* between Gemini and Claude (*P* = .021). The 2-way ANOVA results showed significant interaction effects between model type and prompt series (Figure 2; *P* < .001, F-value = 6.21). Claude generally performed consistently across the prompt series, whereas Gemini had more variability. ChatGPT4o performed well in *Randomized* and *Persona* series but less so in the *Standard* series.

For models and evaluation metrics, Gemini consistently scored lower on *Relevance* across all models, whereas Claude and ChatGPT4o had higher scores. The models exhibited similar trends in *Clarity* and *Relevance*, with Claude generally leading in performance. In terms of prompt series and evaluation metrics, the *Standard* and *Persona* series generally resulted in higher scores across metrics, in contrast to the *Standard Randomized* series performing lower.

The Shapiro-Wilk test indicated that the data did not follow a normal distribution for any of the performance metrics. Kruskal-Wallis tests were used due to non-normality. Significant differences were found for *Empathy/Tone* (*P* = .001), reference to *Evidence* (*P* < .001), and internal *Consistency* (*P* < .001) between models and prompt series.

**Figure 3.**
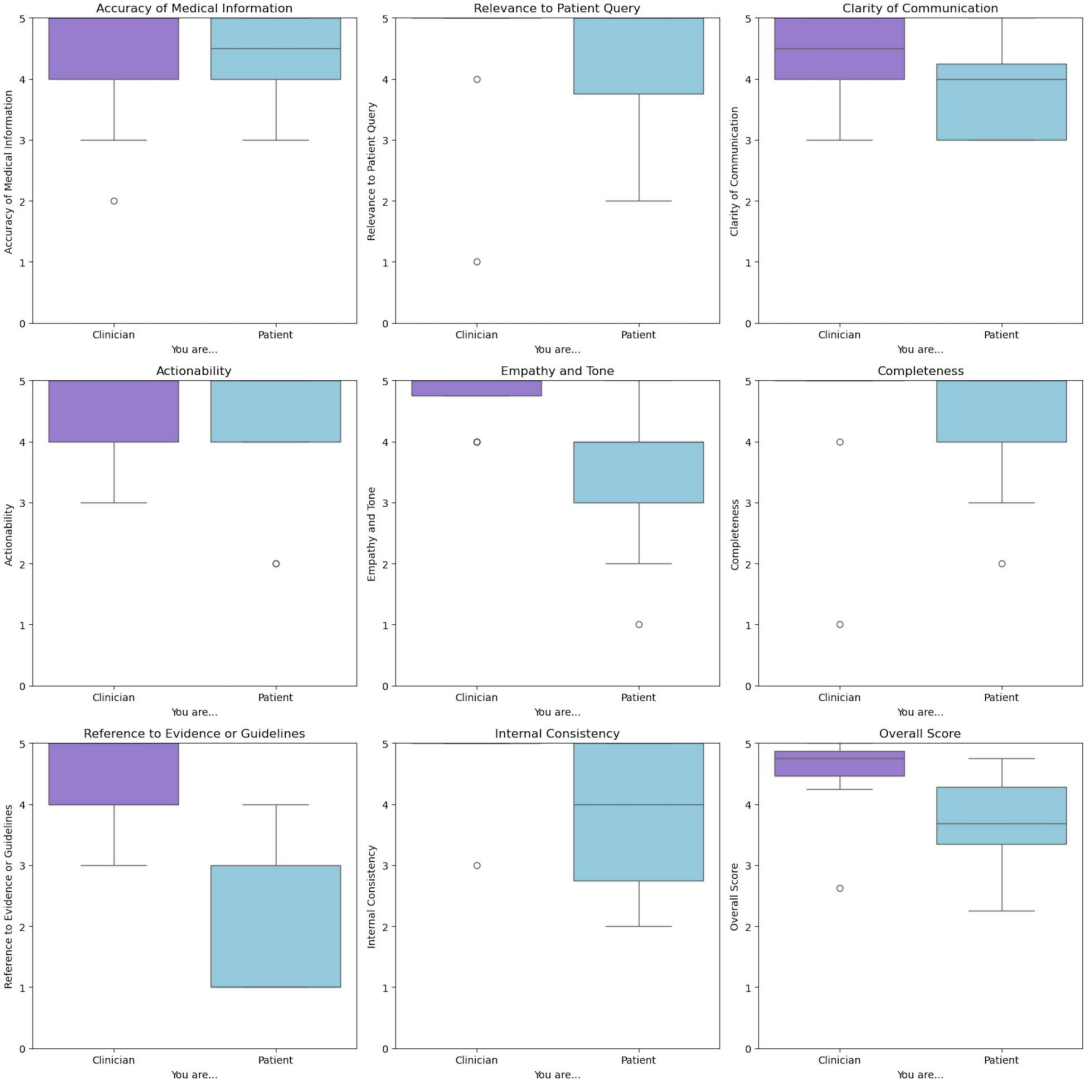
Average score per metric by the clinician and patient rater.

There are notable differences in the distribution of scores between the 2 raters across the same set of performance metrics, including the average overall score (Figure 3, Appendix Table S5). For most metrics, the patient tended to give slightly lower scores compared to the neuro-oncologist, particularly in metrics like *Clarity*, *Empathy/Tone*, and *Evidence*. The pair were most similar on their rating of *Accuracy* and *Actionability* metrics but differed in their rating of *Evidence*, *Empathy*, *Completeness*, and *Relevance*. Gwet’s AC1 calculation demonstrated that *Relevance* (AC1 = 0.53), *Completeness* (AC1 = 0.43), and *Clarity* (AC1 = 0.40) had the most cohesion across raters, though only *Consistency* (AC1 = 0.33, *P* = .016) showed statistically significant agreement. *Evidence* (AC1 = −0.23, *P* = .006) and *Empathy/Tone* (AC1 = 0.00, *P* = .002) exhibited the least agreement between raters, both significantly different from chance. Intraclass Correlation Coefficients (ICCs) were calculated to assess the reliability of their ratings: the patient ICC was 0.523, while the neuro-oncologist ICC was 0.618, indicating both had moderate reliability as raters. The Kruskal-Wallis test results for differences in ratings across different models and prompt series showed no statistically significant differences for any of the metrics.

## Discussion

New AI tools have potential to aid patients by providing additional avenues for inquiry and understanding. We found no significant differences in the variability of ratings between models or prompt series. However, significant interaction effects were found between model type and prompt series, indicating that the performance of the models was influenced by the specific combination of these factors. Claude 3 Opus and ChatGPT 4o models performed most consistently across metrics, particularly in the *Standard Order* series (where patient questions were presented sequentially as they related to the clinical note) and *Persona* series (same as the *Standard* series, but prefixed with a Persona instruction, “*You are an expert oncologist…*”). *Standard* and *Persona* series scored high in *Accuracy*, *Relevance*, and *Clarity*. Gemini 1.5 showed more variability, with lower scores in metrics such as *Reference* and *Evidence.* Post-hoc analysis highlighted differences in *Accuracy* and *Relevance* between certain models, particularly favoring Claude and ChatGPT4o over Gemini.

Collectively, the findings from this proof-of-concept study suggest that all models demonstrated some level of capability in responding to patient queries, however, the utility varied depending on the model used, the prompt series applied, and the evaluator’s perspective on a particular criterion. These results highlight the importance of considering both technical performance and human factors in the evaluation of LLMs for clinical applications, and understanding that different models may have different benefits for different types of queries.

Historically, healthcare often adopted a paternalistic stance regarding patients’ access to and engagement with their medical information.^33^ Clinical notes serve multiple purposes—as reminders for clinicians, as documentation for billing, as reference material for patients—so increased attention to the goal of the user matters. Studies in the domain of health services research have shown that patients often forget much of what is said in clinical visits; this misremembering worsens when a patient is receiving “bad news.”^34^ LLMs have the potential to bridge critical gaps in patient care by serving as an asynchronous support during “in-between” moments when patients are managing care independently.^34–36^

Previous studies have highlighted evaluations of *Empathy/Tone* and *Accuracy* when interacting with LLMs or AI tools on medical topics based on prompts designed by clinicians and researchers, however little attention was given to goals of the user (patients’) interaction with the LLM.^35^ For example, patient users may not prioritize the *Empathy/Tone* in an LLM response as long as they receive factual answers in response to their questions. This study showed *Accuracy* was assessed as reasonable by the patient and clinician rater across most models, whereas other users may primarily use LLMs to help assess *their own understanding* of an open note, or to help plan for a clinician follow up visit—such as demonstrated in early studies of “our notes.”^36^ Future LLM research should specifically evaluate criteria as it relates to the goals of the patient, especially as a growing number of consumers express concerns about AI.^27^

A proactive approach to patient education about LLMs will require a cultural shift away from discouraging patients from conducting their own search for information,^37^ and rather toward helping patients better navigate the information offered by generative AI tools.^38^^,^^39^ Discouraging patients from using AI may face legal challenges or accusations as being an infringement on free speech.^28^ Moving forward, health systems could play an active role in patient use of AI by offering “prompting suggestions” tailored for patients who express interest in using LLMs. By doing so, healthcare providers can ensure patients are equipped with the tools necessary to engage in informed, constructive online health conversations or searches.^40–42^ While clinicians should not be expected to know all details about LLMs, they should be made more aware of the range of variability among them, just as it is reasonable to expect clinicians to generally understand there are differences in medical software or medical devices. Recognizing that there are differences in LLMs, and that not all AI nor all LLMs are the same, is key.

This study did not explicitly use memory-related features—for example, the models did not retain knowledge of past queries—however this should be explored in subsequent research. Memory-related features may allow for more personalization, continuity, context-awareness, and tailored recommendations or language level-setting that would benefit patients over time.

Regulatory agencies already impose transparency requirements on generative AI as part of as part of the Assistant Secretary for Technology Policy and Office of the National Coordinator for Health Information Technology (ASTP/ONC)’s “AI assurance labs”^43^^,^^44^ ASTP/ONC could also suggest collaborations with patient users of health AI in the co-development of ethical frameworks to mitigate inevitable cases of misinformation sparked through hallucinations.^45^ These initiatives could guide how health systems work with, rather than against, patients in their own use of AI technologies.^46^

### Limitations

Only a single clinical note was evaluated by the clinician who wrote it and the patient on whom the note is based. The clinician and patient rater have an existing clinical relationship, but they assessed the note individually. Future studies should evaluate multiple types of notes by including more evaluators without direct knowledge of the encounter covered in the note, using the same prompt series as models from this proof-of-concept study (Appendix S3). One challenge for reproducibility with LLMs is the nature of LLMs, such that the same inputs to the same models may result in slightly varying outputs. Reproduced efforts should not expect identical word output but generally similar outputs. Future studies should incorporate an assessment of the goals of the patient or user who is creating the prompts and using the LLM. For example, the prompts used in this study did not specifically prompt for evidence-based evaluation, yet this was quantified as part of the evaluation rubric, which correlates with this scoring lower compared to other metrics. Future studies should consider prompts that include evidence-based requests and/or the implication of choosing models with features enabled for search and access to an actual evidence base, rather than an LLM-only generated output.

## Conclusion

In this proof-of-concept study, a neuro-oncologist and their patient evaluated the performance of 3 commercially available LLMs in responding to patient queries based on a single progress note across multiple criteria. Incorporating a Persona instruction into a prompt significantly enhanced all LLMs’ performance in eliciting an empathetic yet actionable response. These findings underscore the potential of LLMs to augment patient understanding of clinical notes, but also highlight the importance of prompt design and model selection. As AI continues to evolve, future research should explore LLM performance across more diverse clinical contexts, from both patient and clinician perspectives, and the development of prompting tools to support patient users of LLMs.

## Supplementary Material

ooaf021_Supplementary_Data

## Data Availability

The data underlying this article are available in the article and in its online Supplementary Material (Appendices S1-S7).
