# Supplementary material for: A proof-of-concept study for patient use of open notes with large language models
Source: JAMIA Open. 2025 Apr 9;8(2):ooaf021. doi: 10.1093/jamiaopen/ooaf021 (PMC11980777; doi:10.1093/jamiaopen/ooaf021)

**Appendix Table of Contents:**

- **A (Chart note): The note used for this analysis was generated in the Epic EHR and retrieved using the MyChart patient portal**
- **B (Table): Questions generated and revised as prompt series for the LLMs**
- **C (Table): A comparison of this study’s evaluation rubric vs. PDQI-9 metrics**
- **D-1 (Table): The average scores for each chart note across all performance metrics, plus the overall average score, based on randomized order as presented to raters**
- **D-2 (Table): The average scores for each chart note across all performance metrics, in order by the computed overall average score**
- **E (Table): Computed average scores by rubric metric across all models and prompt series**
- **F (Figure): Visualization of interaction between large language model and rubric evaluation metrics to highlight model performance metrics**

**Appendix A:** **The note used for this analysis was generated in the Epic EHR and MyChart patient portal**

**Name: XXX | DOB: DD/MM/YYYY | MRN: XXX | PCP: XXX MD | Legal Name: XXX**

**A Note to Patients:** Symptoms are concisely summarized to inform treatment recommendations. For reasons of privacy and brevity, this note does not attempt to capture all experiences that were discussed.

Progress Notes

XXX at 9/27/2021 1:00 PM

Video visit scheduled for 10/06/2021. Confirmed w/ pt

XXX at 9/27/2021 1:00 PM

I performed one or more of the following activities: reviewed the patient's records and tests, obtained history, placed orders, coordinated patient care, and/or communicated with other healthcare professionals, the patient, family, or caregiver for a total of 32 minutes on date 09/30/21. This is directly related to an in person or video visit (Evaluation and Management service) I personally provided on date 09/27/21.

Tumor type: Diffuse astrocytoma, grade 2 with gemistocytic features

Location: Left parietal

Age at diagnosis: 29 year-old left-handed female (writes with L hand)

Treatment History:

1. 07/25/2008: Presented with generalized seizure. Imaging revealed left parietal mass

2. 09/17/2008: Resection of left parietal mass by Dr. XXX in Sacramento. Pathology:grade 2 astrocytoma with gemistocytic features

3. 02/06/2009: Further resection of left parietal mass with new enhancement. Pathology:recurrent grade 2 astrocytoma with gemistocytic features

4. 02/2009 - 04/2011: 24 cycles of adjuvant temozolomide

5. Followed

I presented the patient's case at our Tumor Board on 09/30/21 for review. While there has been minimal change over the short term, over the longer term there has been clear increased bulkiness of FLAIR abnormality lateral to the cavity, consistent with slow tumor progression. The consensus of the group was that surgical debulking was recommended as a first step.

I spoke with the patient and her husband by phone, and let them know. They would like to proceed with neurosurgical consultation, and I will refer them to Dr. XXX for that. 99358 for 30-74 minutes total non-face-to-face time

XXX at 9/27/2021 1:00 PM

**NEURO-ONCOLOGY CLINIC NOTE**

**DATE OF VISIT:** 09/27/21

**DATE OF LAST VISIT:** 08/12/21

XXX is a 42 y.o., woman with the following tumor history:

Tumor type: Diffuse astrocytoma, grade 2 with gemistocytic features

Location: Left parietal

Age at diagnosis: 29 year-old left-handed female (writes with L hand)

Treatment History:

1. 07/25/2008: Presented with generalized seizure. Imaging revealed left parietal mass

2. 09/17/2008: Resection of left parietal mass by Dr. XXX in XXX. Pathology: grade 2 astrocytoma with gemistocytic features

3. 02/06/2009: Further resection of left parietal mass with new enhancement. Pathology:recurrent grade 2 astrocytoma with gemistocytic features

4. 02/2009 - 04/2011: 24 cycles of adjuvant temozolomide

5. Followed

**INTERVAL HISTORY:**

Patient presents with her husband

Since her last clinic visit, she has been feeling well, without headaches, seizures or other focal neurological complaints.

**PAST MEDICAL HISTORY**

**Past Medical History:**

Diagnosis

Date

- At risk for cancer
  *Genetic Cancer Risk per pt*
- Brain tumor (CMS code)
- Cancer (CMS code)
- Easy bruising
- Eczema
- Headache
- History of placement of ear tubes
  *In childhood*
- Restless leg syndrome
- Seasonal allergies
- Seizures (CMS code)
- Sensorineural hearing loss
  *Left*

| **MEDICATIONS: Medications the patient states to be taking prior to today's encounter.** |  |  |
| --- | --- | --- |
| Medication | Sig |  |
|  | clonazePAM (KLONOPIN) 1mg rapid dissolve tablet | Take 1 mg by mouth once as needed (As Needed). |
| • | lacosamide (VIMPAT) 200 mg tablet | Take 1 pill in the morning and 2 pills in the evening for a total dose of 600 mg per day. |
| • | levETIRAcetam (KEPPRA) 750mg tablet | Take 2 tablets (1500mg) twice daily. (Patient taking differently: 750 mg Take 2 tablets (1500mg) twice daily.) |
| • | levonorgestrel (MIRENA) 20mcg/24 hr (5 years) IUD | 1 each by Intrauterine route once. |
| • | LORazepam (ATIVAN) 1 mg tablet | Take 1 tablet (1 mg total) by mouth every 8 (eight)hours as needed (Seizures) C71.9, 30 day supply |
| • | rOPINIRole (REQUIP) 1 mg tablet | Take 1 mg by mouth daily |
| • | SUMAtriptan (IMITREX) 50 mg tablet | Take 1 tablet (50 mg total) by mouth once as needed for Migraine (May repeat dose after 2 hoursx1 if headache recurs) for up to 1 dose |

**ALLERGIES**

Allergies as of XXX - Review complete XXX

Allergen: Lamotrigine

Reaction: Rash

Noted: 05/23/2017

**REVIEW OF SYSTEMS**: Over the last 30 days, the patient also attests to joint pain.

Patient denies chest pain, shortness of breath, new skin lesions. A complete 14 point review of systems was performed and pertinent positives and negative recorded in theHPI.

**PHYSICAL EXAMINATION:**

**Vital Signs:** BP 123/60 | Pulse 62 | Temp 36.6 °C (97.9 °F) (Oral) | Resp 16 | Ht 170.2 cm (5' 7") | Wt 73.1 kg (161 lb 3.2 oz) | SpO2 100% | BMI 25.25 kg/m²

**KPS:** 100

**ECOG:** 0

**General Exam:** She is a well appearing female in no acute distress.

**Head and neck:** Well-healed craniotomy scar without drainage or erythema. Oropharynx clear without lesions.

**Neurologic Exam:** Appropriate attention, memory, and affect. Speech fluent without dysarthria or aphasia - specifically naming, repetition, and comprehension were intact.

**Cranial Nerves:** Extraocular movements intact without nystagmus. Visual fields full to confrontation. Face symmetric with sensation slightly reduced to light touch on the R. Hearing symmetric to finger rub. Tongue midline. Uvula and palate symmetric.

**Motor Exam:** No drift. Normal and symmetric bulk and tone. No orbiting. Symmetric finger tapping. Strength 5/5 throughout.

**Sensation:** Mildly reduced to light touch in the upper and lower extremities on the R side.

**Coordination:** No dysmetria on finger-to-nose testing.

**Stance and Gait:** Normal gait. Able to walk on toes, heels, and tandem without difficulty.

**IMAGING**: I personally reviewed the most recent MRI with the patient and family from 09/27/21, and compared it to previous MRI from 07/09/21, 07/10/20 and 08/21/18 demonstrating gradual increase in bulkiness of FLAIR abnormality lateral to the surgical cavity, though minimal if any chance since July. This does unfortunately look consistent with gradual tumor progression.

**Report**

From 09/27/21

Compared to multiple priors

*Impression:*

*No significant change compared to 7/9/2021, however nonenhancing FLAIR signal most notably at the superolateral resection margin has slowly progressed since more remote priors dating back to 2017.*

**ASSESSMENT AND PLAN**:

In summary, Ms. XXX is a 42 y.o. year old woman with grade 2 astrocytoma who is neurologically nonfocal but who has evidence of gradual tumor progression over years.

IMAGING SCORE: -2

NEUROLOGICAL SCORE: 0

STEROID SCORE: 0

OVERALL TUMOR ASSESSMENT: -2

1. Grade 2 astrocytoma: I do think the tumor recurrence would be surgically resectable, and I would like to present her case at our Tumor Board this week for discussion to confirm if that is the recommendation. If so, and if there is gross-total resection of tumor that remains grade 2, then we may be able to simply monitor her going forward. If there is visible residual, then it may make sense to either treat with an IDH-inhibitor or to consider chemoradiation. She is open to surgery if felt feasible.

- All questions of XXX and her family were answered to the best of my ability. They have our contact information and will call with further questions.

I spent a total of 49 minutes on this patient's care on the day of their visit excluding time spent related to any billed procedures. This time includes time spent with the patient as well as time spent documenting in the medical record, reviewing patient's records and tests, obtaining history, placing orders, communicating with other healthcare professionals, counseling the patient, family, or caregiver, and/or care coordination for the diagnoses above.

Patient Instructions

XXX at 9/27/2021 1:00 PM

Good to see you, though I'm sad that it looks like there's tumor growth. As discussed, I'll call you after Tumor Board on Thurs to let you know the recommendations.

Take care, XXX

MyChart® licensed from Epic Systems Corporation © 1999 - 2023

**Appendix B: Questions generated and revised as prompt series for the LLMs**

| **Original Questions List (DL)** | **Modified Questions List (FR, EM, DL)** | **Notes about changes** |
| --- | --- | --- |
| What is FLAIR abnormality? | What is FLAIR abnormality? How does that differ from regrowth? | Add: “how differs from regrowth” |
| How much is the clear increased bulkiness? | How large is the area of ‘clear increased’ bulkiness - how has this compared to my previous recurrence? | Clarifying language |
| Which area is the ‘lateral’ part of the cavity? What part of my brain would that affect? What part of my body would that influence? | Which area is the ‘lateral’ part of the cavity? What part of my brain would that affect? What part of my body would that influence? | (No change) |
| How does surgical debulking compare to resection process? How is it different? | How does surgical debulking compare to resection process? How is it different? Is the recovery time expected to be the same for surgical debulking as it was for resection? Does it change given this would be my third surgery? | Combined two questions |
| Is the recovery time expected to be the same for surgical debulking as it was for resection? Does it change given this would be my third surgery? |  |  |
|  | If surgery is the option moving forward - how soon should I have it? | Added question |
| How likely is my tumor to again be grade 2, given my last 2 resections were both grade 2 astrocytoma with gemistocytic features? | How likely is my tumor to again be grade 2, given my last 2 resections were both grade 2 astrocytoma with gemistocytic features? | (No change) |
|  | Even if there is not residual that is visible post-op, might an IDH inhibitor be considered anyway, given the history of low grade recurrences? | Added question |
|  | Would chemoradiation still be TMZ or would it be different? | Added question |
|  | Would you expect chemoresistance to TMZ given the history of use? | Added question |
| In the meantime, should I do anything different about my joint pain? | In the meantime, should I do anything different about my joint pain? | (No change) |
| What should I be watching for in terms of symptoms or things of concern? | What should I be watching for in terms of symptoms or things of concern? | (No change) |

**Appendix C: A comparison of this study’s evaluation rubric vs. PDQI-9 metrics**

| **This Study's Rubric** | **Similarity** | **PDQI-9 Rubric Items** | **Present in Our Study** | **Present in PDQI-9** |
| --- | --- | --- | --- | --- |
| Accuracy of Medical Information | Yes | Accurate | Yes | Yes |
|  | Yes | Up-to-date | Yes | Yes |
| Relevance to Patient Query | Yes | Useful | Yes | Yes |
| Clarity of Communication | Yes | Comprehensible | Yes | Yes |
|  | Yes | Organized | Yes | Yes |
| Completeness | No | Thorough | Yes | Yes |
| Empathy and Tone | No | -- | Yes | **No** |
| Actionability* | No | -- | Yes | **No** |
| -- | No | Succinct | **No** | Yes |
| -- | No | Synthesized | **No** | Yes |
| Internal Consistency | Yes | Internally Consistent | Yes | Yes |

** Actionability: actions a patient may take in response to information provided*

**Appendix D-1: The average scores for each chart note across all performance metrics, plus the overall average score, based on randomized order as presented to raters**

| **Chart Note** | **Accuracy of Medical Information** | | **Relevance to Patient Query** | | **Clarity of Communication** | | **Actionability** | | **Empathy and Tone** | | **Completeness** | | **Reference to Evidence or Guidelines** | | **Internal Consistency** | | **Overall Score** | |  |
| --- | --- | --- | --- | --- | --- | --- | --- | --- | --- | --- | --- | --- | --- | --- | --- | --- | --- | --- | --- |
| Claude,  Standard | | 5 | | 5 | | 4 | | 4.5 | | 4 | | 5 | | 3 | | 5 | | 4.44 | |
| Gemini,  Persona | | 3.5 | | 3.5 | | 3.5 | | 4.5 | | 4.5 | | 4.5 | | 2.5 | | 3.5 | | 3.75 | |
| Gemini,  Randomized Persona | | 4 | | 4 | | 4.5 | | 4.5 | | 4.5 | | 4.5 | | 2.5 | | 4 | | 4.06 | |
| Gemini,  Standard | | 4.5 | | 5 | | 5 | | 5 | | 4.5 | | 5 | | 2.5 | | 5 | | 4.56 | |
| Claude,  Persona | | 4 | | 5 | | 4 | | 5 | | 5 | | 5 | | 3.5 | | 5 | | 4.56 | |
| Claude,  Random | | 4.5 | | 5 | | 3 | | 4.5 | | 3 | | 4.5 | | 3 | | 4.5 | | 4 | |
| GPT4o,  Random | | 4.5 | | 4.5 | | 4 | | 3.5 | | 4 | | 4.5 | | 3 | | 4.5 | | 4.06 | |
| GPT4o,  Persona | | 5 | | 5 | | 5 | | 5 | | 4.5 | | 5 | | 4.5 | | 5 | | 4.88 | |
| Claude,  Randomized Persona | | 5 | | 5 | | 4 | | 4.5 | | 4.5 | | 5 | | 4 | | 4.5 | | 4.56 | |
| GPT4o,  Randomized Persona | | 5 | | 5 | | 4 | | 4.5 | | 3 | | 5 | | 3.5 | | 5 | | 4.38 | |
| Gemini,  Random | | 3 | | 2.5 | | 4.5 | | 4 | | 4 | | 2 | | 2 | | 2.5 | | 3.06 | |
| GPT4o,  Standard | | 4 | | 3.5 | | 4 | | 3.5 | | 3.5 | | 3.5 | | 3 | | 3.5 | | 3.56 | |

**Appendix D-2: The average scores for each chart note across all performance metrics, in order by the computed overall average score**

| **Chart Note** | **Accuracy of Medical Information** | | **Relevance to Patient Query** | | **Clarity of Communication** | | **Actionability** | | **Empathy and Tone** | | **Completeness** | | **Reference to Evidence or Guidelines** | | **Internal Consistency** | | **Overall Score** | |  |
| --- | --- | --- | --- | --- | --- | --- | --- | --- | --- | --- | --- | --- | --- | --- | --- | --- | --- | --- | --- |
| GPT4o,  Persona | | 5 | | 5 | | 5 | | 5 | | 4.5 | | 5 | | 4.5 | | 5 | | 4.88 | |
| Gemini,  Standard | | 4.5 | | 5 | | 5 | | 5 | | 4.5 | | 5 | | 2.5 | | 5 | | 4.56 | |
| Claude,  Persona | | 4 | | 5 | | 4 | | 5 | | 5 | | 5 | | 3.5 | | 5 | | 4.56 | |
| Claude,  Randomized Persona | | 5 | | 5 | | 4 | | 4.5 | | 4.5 | | 5 | | 4 | | 4.5 | | 4.56 | |
| Claude,  Standard | | 5 | | 5 | | 4 | | 4.5 | | 4 | | 5 | | 3 | | 5 | | 4.44 | |
| GPT4o,  Randomized Persona | | 5 | | 5 | | 4 | | 4.5 | | 3 | | 5 | | 3.5 | | 5 | | 4.38 | |
| Gemini,  Randomized Persona | | 4 | | 4 | | 4.5 | | 4.5 | | 4.5 | | 4.5 | | 2.5 | | 4 | | 4.06 | |
| GPT4o,  Random | | 4.5 | | 4.5 | | 4 | | 3.5 | | 4 | | 4.5 | | 3 | | 4.5 | | 4.06 | |
| Claude,  Random | | 4.5 | | 5 | | 3 | | 4.5 | | 3 | | 4.5 | | 3 | | 4.5 | | 4 | |
| Gemini,  Persona | | 3.5 | | 3.5 | | 3.5 | | 4.5 | | 4.5 | | 4.5 | | 2.5 | | 3.5 | | 3.75 | |
| GPT4o,  Standard | | 4 | | 3.5 | | 4 | | 3.5 | | 3.5 | | 3.5 | | 3 | | 3.5 | | 3.56 | |
| Gemini,  Random | | 3 | | 2.5 | | 4.5 | | 4 | | 4 | | 2 | | 2 | | 2.5 | | 3.06 | |

**Appendix E: Computed average scores by rubric metric across all models and prompt series**

| **Metric** | **Mean** | **Std. Dev** | **Min** | **25th Percentile** | **Median** | **75th Percentile** | **Max** |
| --- | --- | --- | --- | --- | --- | --- | --- |
| Accuracy of Medical Information *(Accuracy)* | 4.08 | 0.91 | 2 | 3.75 | 4 | 5 | 5 |
| Relevance to Patient Query *(Relevance)* | 4.14 | 1.13 | 1 | 3.75 | 5 | 5 | 5 |
| Clarity of Communication *(Clarity)* | 4.08 | 0.77 | 3 | 3.75 | 4 | 5 | 5 |
| Actionability | 3.81 | 1.31 | 1 | 3 | 4 | 5 | 5 |
| Empathy and Tone *(Empathy/Tone)* | 3.53 | 1.28 | 1 | 3 | 4 | 5 | 5 |
| Completeness | 3.72 | 1.49 | 1 | 2 | 4 | 5 | 5 |
| Reference to Evidence or Guidelines *(Evidence)* | 2.69 | 1.47 | 1 | 1 | 3 | 4 | 5 |
| Internal Consistency *(Consistency)* | 4.06 | 1.26 | 1 | 3 | 5 | 5 | 5 |

**Appendix F:** **Visualization of interaction between large language model and rubric evaluation metrics to highlight model performance metrics**


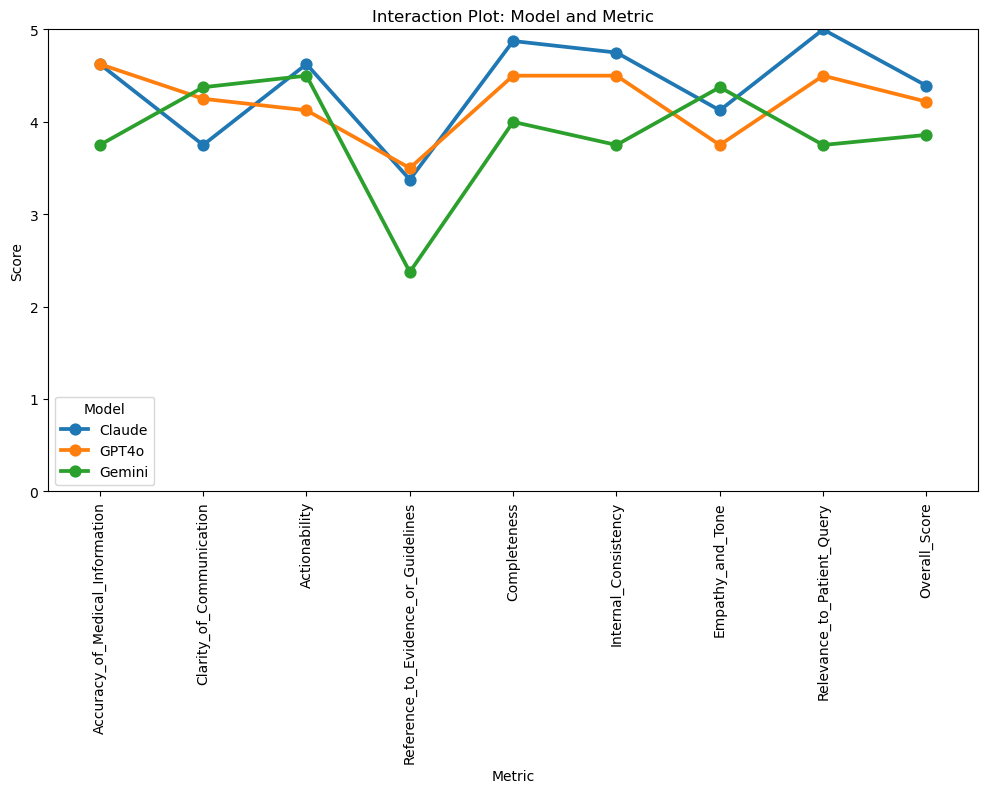

Supplement: ooaf021_Supplementary_Data [file ooaf021_supplementary_data.docx]
